# Phytic Acid and Transporters: What Can We Learn from *low phytic acid* Mutants?

**DOI:** 10.3390/plants9010069

**Published:** 2020-01-05

**Authors:** Eleonora Cominelli, Roberto Pilu, Francesca Sparvoli

**Affiliations:** 1 Institute of Agricultural Biology and Biotechnology, Consiglio Nazionale delle Ricerche, Via E. Bassini 15, 20133 Milan, Italy; sparvoli@ibba.cnr.it; 2 Department of Agricultural and Environmental Sciences—Production Landscape, Agroenergy Università degli Studi di Milano, Via G. Celoria 2, 20133 Milan, Italy; salvatore.pilu@unimi.it

**Keywords:** phytic acid, low phytic acid mutants, MRP transporter, ABCC transporter, SULTR transporter, Pht, phosphate transporter, sulfate transporter

## Abstract

Phytic acid has two main roles in plant tissues: Storage of phosphorus and regulation of different cellular processes. From a nutritional point of view, it is considered an antinutritional compound because, being a cation chelator, its presence reduces mineral bioavailability from the diet. In recent decades, the development of low phytic acid (*lpa*) mutants has been an important goal for nutritional seed quality improvement, mainly in cereals and legumes. Different *lpa* mutations affect phytic acid biosynthetic genes. However, other *lpa* mutations isolated so far, affect genes coding for three classes of transporters: A specific group of ABCC type vacuolar transporters, putative sulfate transporters, and phosphate transporters. In the present review, we summarize advances in the characterization of these transporters in cereals and legumes. Particularly, we describe genes, proteins, and mutants for these different transporters, and we report data of in silico analysis aimed at identifying the putative orthologs in some other cereal and legume species. Finally, we comment on the advantage of using such types of mutants for crop biofortification and on their possible utility to unravel links between phosphorus and sulfur metabolism (phosphate and sulfate homeostasis crosstalk).

## 1. Introduction

Phytic acid (PA), chemically *myo*-inositol-1,2,3,4,5,5-hexakisphosphate, is the major form of phosphorus (P) storage in seeds (up to 85% of total P) and in other plant organs, such as pollen, roots, tubers, and turions. However, PA is not only an important molecule for P storage but, together with its precursors (lower InsPs and *myo*-inositol) and its derivative molecules (InsP7 and InsP8 inositol pyrophosphates), it is involved in the regulation of different cell signaling and plant processes in vegetative tissues, such as abiotic and biotic stress response, storage and polar transport of auxin, P homeostasis, photomorphogenesis, chromatin modification, and remodeling and mRNA nuclear export [1].

In seeds, where P amounts may even be 1000-fold higher than those detected in vegetative tissues, PA is accumulated during development, reaching a plateau at the end of the “cell expansion phase” [2,3]. PA is synthesized in the cytosol through two different routes: (i) The lipid-independent pathway, the most used in the seed, consisting of the sequential phosphorylation of the 6-carbon *myo*-inositol and soluble inositol phosphates (InsPs), and (ii) the lipid-dependent pathway, using precursors that include phosphatidylinositol (PtdIns) and PtdIns phosphates. PA is transferred from the cytosol to the vacuole where it is accumulated into globoids, spherical inclusions found within protein bodies [4,5,6,7]. Interestingly, the amount and distribution of PA in different seed/grain portions vary among different species. In cereals, there are differences between *Zea mays* L. (maize) kernels, where PA is mainly present in the embryo and scutellum, and the small grains of *Hordeum vulgare* L. (barley), different *Triticum* (wheat) species and *Oryza sativa* L. (rice), where 80% of PA is stored in the aleurone and bran (maternal teguments) and only a limited amount accumulates in the embryo [8]. However, in legumes, more than 95% seed PA is accumulated in the cotyledons [9]. During germination, phytases degrade PA and in this way, P is remobilized to support seedling growth [10]. Due to its high negative charge at physiological pH (~6–7), PA easily precipitates in the form of phytate salts binding cations, such as iron, zinc, potassium, calcium, magnesium, some of them (mainly iron and zinc) important from a nutritional point of view, in this way reducing their bioavailability. Only ruminants are able to degrade PA, due to the presence of microbial phytases in their digestive tracts, while for monogastric animals, including humans, mainly in those populations whose diet is largely based on staple crops, the presence of PA decreases seeds’ nutritional value [11,12]. Moreover, as undigested PA is excreted by non-ruminants, such as swine, fowl, and fish, the supplementation of feed with nutrient P is a common practice, in order to provide for an animal’s nutritional requirement. In this way P concentrations increase in manure, consequently in soils, finally contributing to P pollution in runoff water [13]. Hence, PA is considered an antinutrient and in recent decades, many efforts were spent to isolate and develop low phytic acid (*lpa*) crops, in which a 45–90% reduction of PA was achieved [1]. Unfortunately, it was shown that the reduction in PA content may affect plant growth, plant stress response and seed development and germination, thus limiting the efficacy of the introgression of the *lpa* trait into breeding programs [14]. The negative pleiotropic effects of the *lpa* mutations depend on the previously mentioned important roles PA has in different regulatory processes.

Hence, it is very important to identify the best strategy in order to specifically decrease PA content in the seeds without affecting plant and seed performance and possibly contribute to reducing the environmental impact. The *lpa* mutations so far isolated can be classified into three classes, depending on the step of the biosynthetic pathway or transport they affect: (i) Mutations altering the MIPS activity, the first steps of the biosynthetic pathway (from glucose 6-P to *myo*-inositol[3]-monophosphate), (ii) mutations affecting the following phosphorylation of the InsP6 pathway (from *myo*-inositol[3]-monophosphate to PA), (iii) mutations perturbing the final transport of PA.

Only mutants belonging to class (ii) accumulate InsPs intermediates. The mutations belonging to the (i) and (iii) classes induce a decrease of PA amount, accompanied by a molar equivalent increase of inorganic phosphate (P_i_) in the homozygous mutants. Moreover, they are usually perturbed in different branches of the biosynthetic pathway common to PA and other compounds (e.g., galactinol, raffinose, stachyose, and ascorbic acid). Mutants in three classes of transporters have been characterized for their *lpa* phenotype, affected in: (i) A specific group of ABCC type vacuolar transporters [15], orthologues to the *Arabidopsis thaliana* (L.) Heinh AtMRP5 (also referred to as AtABCC5) [16,17], also known as multidrug resistance-associated proteins (MRPs), (ii) putative sulfate transporters, orthologues of the Arabidopsis AtSULTR3;3 [18,19] and AtSULTR3;4 proteins [20]; (iii) the rice OsPht1;4 phosphate transporter [21,22]. Only in the case of an ABCC transporter was it shown that the protein is able to actually transport PA [16].

In this review we will discuss the advances in the characterization of PA-MRP, PA-SULTR, and OsPht1;4 transporters, and of the corresponding mutants described so far in cereals and legumes. Particular emphasis will be given to the reported differences among cereals and legumes of *lpa* mutant phenotypes in the PA-MRP genes, depending on the presence of one or more partially redundant copies of these genes and to their tissue-specific expression. Moreover, we identified in silico the putative orthologs of PA-SULTR in species of interest for the isolation of *lpa* mutants. We will also discuss the advantages of these mutants for crop biofortification. Furthermore, we will highlight how the study of these mutants may help to elucidate phosphate and sulfur metabolism, and the possible roles that the transporters described here may play.

## 2. PA-MRP Transporters

MRP proteins are transmembrane transporters involved in several functions, such as organic ions transport, xenobiotic detoxification, oxidative stress tolerance, and transpiration control [23]. The first evidence of the involvement of an MRP-type ABC transporter in PA transport was reported for the maize ZmMRP4 protein from the analysis of the insertional *lpa1* mutant [24]. *ZmMRP4* gene is orthologous to *AtMRP5*, which had already been characterized some years ago as an anion transporter involved in root growth, lateral root formation, regulation of stomatal movement, guard cell hormonal signaling, and water use efficiency [25,26,27], aspects not immediately attributable to PA transport. The biochemical demonstration that PA transport was dependent on the presence of a functional MRP transporter in an ATP-dependent manner was given for AtMRP5, showing a very high affinity for PA (maximum reaction velocity -V_max_- values of about 1.6–2.5 µmol min^−1^ mg^−1^ and Michaelis—Mentent constant -*K*_m_- ranging between 263 and 310 nM) and a vacuolar subcellular localization [16].

As summarized in Table 1, other *PA-MRP* genes and the corresponding mutants/transgenics have been hereafter characterized in rice, *Glycine max* (L.) Merr. (soybean), *Phaseolus vulgaris* L. (common bean) and *Triticum aestivum* L. (soft wheat) [28,29,30,31,32,33]. Moreover, the putative *Pennisetum glaucum* (L.) R.Br. (pearl millet) *PA-MRP* gene has recently been described [34].

The main difference between cereals and legumes for which PA-MRPs have been characterized so far is the gene number: While only one gene is present in diploid maize, pearl millet, and rice genomes, and three copies in the hexaploid *Triticum aestivum*, two and three paralogues are present in common bean and soybean, respectively [17,32,33]. The presence of more than one member of the *PA-MRP* genes seems to be a common feature of legumes, for example also in *Medicago truncatula* it is possible to predict two *PA-MRP* genes [35], unlike the situation in other dicotyledons, such as Arabidopsis and *Solanum lycopersicum* L. (tomato) in which only one *PA-MRP* gene was described [16,36]. As discussed below, the gene copy number has a significant influence on the *lpa* mutant phenotypes.

**Figure 1:**
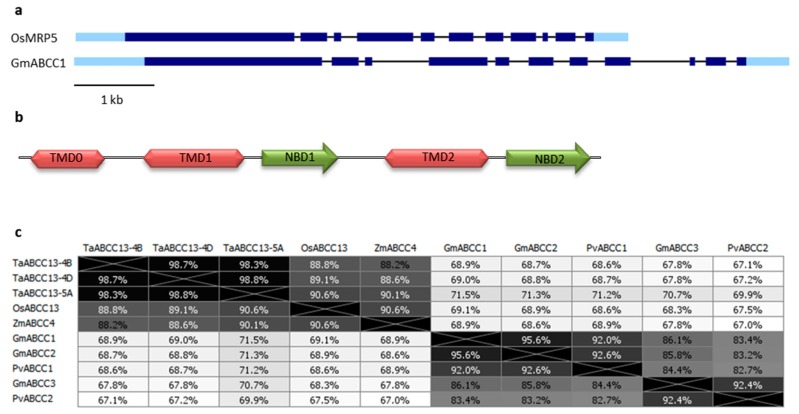
(**a**) Gene structure of *OsMRP5* and *GmABCC1* genes, as an example of a cereal and a legume *PA-MRP* gene, respectively. Light and dark blue rectangles represent UTRs and coding exons, respectively, the black bars correspond to introns. Gene Structure Display Server [46] was used; (**b**) Predicted domains of the PA-MRP protein. The transmembrane domains (TMD) and the nucleotide-binding domains (NBD) are represented in red and green, respectively. The structure of the PA-MRP proteins was previously described [17]; (**c**) Distances between PA-MRP proteins, expressed as a percentage of identity. Phylogenies were constructed with the Geneious Tree Builder tool, using the Jukes—Cantor distance model, neighbor-joining tree build method.

The gene structure of PA-MRP transporters is very highly conserved: All analyzed genes in the present study have 11 exons and 10 introns with very similar lengths, only some differences can be found in the lengths of some introns between cereals and legumes. In Figure 1a, the rice *OsMRP5* and the soybean *GmABCC1* gene schematic representations are given as examples.

PA-MRP proteins are full-length ABC transporters (length from 1501 aa of TaABCC13–4B to 1539 aa of GmABCC1) with three membrane-spanning domains (TMD0, containing five transmembrane α-helices and TMD1, TMD2, each with six α-helices) and two cytosolic nucleotide-binding domains (NBD1 and NBD2, containing the Walker A and B motifs), arranged in the TMD0-TMD1-NBD1_TMD2-NBD2 so-called forward orientation (Figure 1b), as previously described [17]. Although it is not known which specific amino acids are involved in PA transport, a conserved lysine residues stretch, located in the cytosolic loop linking NBD1 and TMD2 and a number of charged amino acid residues (mostly lysine and arginine) found in other conserved stretches in TMD1 and TMD2 have been suggested to be involved in PA transport [17].

PA-MRP protein sequences are phylogenetically very highly conserved among different species, mainly in the TMD and NBD domains, but also outside, particularly among cereals or legumes (Figure 1 and Figure S1). As shown in Figure 1c, the degree of aminoacid identity among different PA-MRP proteins belonging to different species is very high, also between cereals and legumes where it ranges from 67 to 71.5% (similarity between cereals and legumes ranges from 86 to 89%, data not shown).

**Figure 2:**
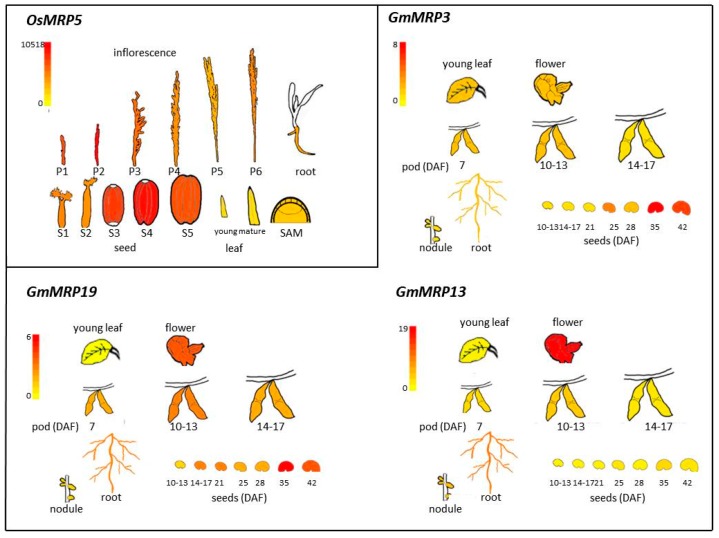
PA-MRP gene expression data in various rice and soybean organs and tissues were obtained from the rice and soybean eFP Browsers [47]. Rice MAS and soybean Severin data sources were used. For rice, the default signal threshold was used, while for the three soybean genes the signal threshold was arbitrarily put to the same value (8.00) in order to compare expression data between different genes.

In Figure 2, pictographic representations of the different organs’ expression patterns of the *OsMRP5* and the soybean *PA-MRP* genes, taken as examples for cereals and legumes, are reported from the rice and soybean eFP Browsers [47]. The rice gene is expressed at high levels in different organs including the caryopsis, as previously reported [41]. The maize ortholog shows a similar expression pattern [24]. The *TaABCC13* genes are expressed in different plant organs, preferentially during grain developmental stages, with the transcript accumulation derived from the B genome the highest one, mainly at 14 days after anthesis [33,48].

The expression pattern of the different legume genes varies. As shown in Figure 2, *GmMRP3* and *GmMRP19* genes are expressed in different organs and highly expressed in seed, particularly at the late stage of development, while the *GmMRP13* gene is mainly expressed in root and flower and at a very low level in seed. As discussed below, when both *GmMRP3* and *GmMRP19* are mutated an *lpa* seed phenotype occurs [30]. It suggests that *GmMRP3* and *GmMRP19* have an important role in PA accumulation and their function is redundant, while *GmMRP13* is not active in the seed.

A similar diversified expression pattern was observed in common bean, where the *PvMRP1* gene, coding for a protein more similar to GmMRP3 and GmMRP19 (Figure 1c and Figure S1), is highly expressed in cotyledons, where its transcript levels continue to increase during seed development, reaching the highest levels at 28 days after flowering (DAF) with a similar kinetics to that reported for the accumulation of PA in the same organ. The *PvMRP2* gene, ortholog of *GmMRP13*, is expressed similarly to *PvMRP1* in vegetative organs, but at no appreciable level in cotyledons. Interestingly, both genes are expressed in root nodules, organs specialized in symbiosis with nitrogen-fixing bacteria, in which the role of PA is still unknown [35]. Recently, a detailed analysis was reported of GUS activity in *Arabidopsis thaliana* and *Medicago truncatula* plants, harboring a promoter sequence of *PvMRP1* and *PvMRP2* genes, fused upstream of the *GUS* reporter gene. The strongest GUS activity, driven by both constructs, in organs other than the seeds was present in the vascular tissues [35]. Similar patterns of reporter gene activity were previously shown in transgenic plants harboring the *AtMRP5* promoter [25] and promoters of different Arabidopsis genes coding for enzymes involved in different steps in PA pathway [49,50,51,52,53,54,55,56]. These data suggest that vascular tissues are an important site for synthesis and transport of PA involved in the regulation of different cellular processes, the so-called “signaling PA” [57].

### lpa Mutants in PA-MRP Transporters

As shown in Table 1, the majority of the *lpa* mutations affecting transporters concern mutations in PA-MRP proteins. Differences exist between cereal and legume *lpa* mutants, with cereal mutants generally affected by more pronounced negative pleiotropic effects mainly due to: (i) The different accumulation of PA in seed/caryopsis compartments, as previously mentioned, (ii) the presence of only one gene coding for a PA-MRP transporter in cereal genomes and more than one in legumes [17,37,38]. As previously discussed, there are similarities between mutants affected in PA biosynthetic genes and in PA transport in the reduction of PA content, accompanied by a molar equivalent increase of P_i_ and the absence of accumulation of InsPs intermediates. For this reason, the first efforts to map the maize *lpa1* mutation suggested that the *myo*-inositol 3-phosphate synthase (*MIPS*) gene coding for the first enzyme of the pathway was mutated [37,38]. This was also corroborated by mapping and expression data, since in maize the *ZmMIPS1S* and the *ZmMRP4* genes map very closely on chromosome 1S, and in mutants affecting *ZmMRP4* the expression of *ZmMIPS1S* is reduced [24,37,38,58]. However, transposon mutagenesis tagging experiments conducted by Shi et al. (2007) demonstrated that *lpa1* gene encodes a multidrug-associated-protein (MRP) named ZmMRP4 (accession number EF586878). As shown in Table 1, different *lpa* mutations were isolated in the maize ZmMRP4 and the rice OsMRP5 PA transporters [4,24,28,37,38,41,58,59,60,61,62,63]. Due to the previously mentioned important roles of PA in different regulatory processes and due to the fact that in these species only a *PA-MRP* gene is present, these mutants display negative pleiotropic effects on plants (stunted vegetative growth) and seeds, such as reduced seed development and weight, low germination rates, making these mutants of limited value to breeders [37,59,64,65,66]. In maize, the most studied model species, four different mutants affecting the *ZmMRP4* locus were isolated: *lpa1-1*, consisting of a point mutation that causes an A1432V substitution in the NBD2 region [24,37], *lpa1-241*, a paramutagenic allele [60] that causes a remarkable variability of expression with a different degree of negative pleiotropic effects depending on its strength [59], *lpa1-7*, whose molecular feature is not known, although the nature of a paramutagenic allele can be excluded [39] and *lpa1-5525,* not yet fully characterized [67]. In the *lpa1-1* mutant, kernel PA was reduced by 66% [37], whilst *lpa1-241* and *lpa1-7* mutants showed the highest reduction in PA with more than 80% [39,59]. All these mutants do not perturb the total P present but are characterized by a five- to ten-fold increase in the amount of free phosphate in the kernel [37,38,39].

In rice, the *Os-XS-lpa2-1* and *Os-XS-lpa2-2* mutations have been isolated at the *OsMRP5* locus [28]. The *Os-XS-lpa2-1* mutant shows a grain PA reduction of about 20% caused by a single base pair substitution mutation in the transmembrane domain TMD2 [41]. In the case of *Os-XS-lpa2-2* the PA reduction is more than 90% due to a 5-bp deletion determining a frame shift causing a premature stop codon at aa 474. The same phenotype was observed in a T-DNA knock outline (4A-02500), demonstrating the important involvement of this gene in PA transport [41]. Unfortunately, in these maize and rice mutants, there is a correlation between the severity of the negative pleiotropic effects and the PA content. In fact, the strongest maize *lpa1-241* and *lpa1-7* mutants and rice *Os-XS-lpa2-2* and *4A-02500* are lethal in the homozygous state, while the other milder mutants (*lpa1-1* and *Os-XS-lpa2-1*) are viable, although showing yield losses compared to wild type [37,39,41,59]. The incapacity to germinate is probably due to the impaired embryo development, mainly because of the displacement of the root primordium and the consequent asymmetry in the body plan, as shown in the maize *lpa1-241* mutant [39,59]. Furthermore, the maize *lpa1-1* mutant and barley *lpa* mutants, such as *Hvlpa1*, *Hvlpa2*, *Hvlpa3,* and *Hv-M955* mutants affected in other genes, are more sensitive to drought stress in the field [68]. The negative pleiotropic effects could be associated with an alteration of the mature root system, as demonstrated in the case of the maize *lpa1-7* mutant [39]. In the latter mutant, other pleiotropic effects associated with the *lpa* mutation have been described, such as reduced carotenoid and chlorophyll content and increased length and trichome density compared with wild type sibling leaves [39].

Another explanation for the lethal phenotype due to the strongest mutations was proposed by Doria and colleagues [62]: They showed that whole *lpa1-241* mutant kernels contained about 50% more free iron associated with a higher content of free radicals than the wild type control. Furthermore, higher production of hydrogen peroxide was found in the embryo of *lpa1-241* grains, particularly in the ones artificially aged. Taken together, these results confirmed that PA is involved in the prevention of oxidative stress in grains, previously only suggested [69,70,71] and considered to be important for the maintenance of the viability of grains [37,72]. Another hypothesis to explain the negative pleiotropic effect associated with mutations affecting the multidrug-associated-protein (MRP) in *lpa* mutants could be that this protein is involved directly or indirectly in the transport of other molecules in addition to PA. In fact, it was observed that the *lpa1-241* mutation, in a genetic background capable of accumulating anthocyanins in the scutellum (embryo tissue), conferred a bluish color in comparison to the reddish wild type control. This alteration was attributed to a defect in the pigment transport in the vacuole, causing a mislocalized accumulation of these pigments in the cytosol, suggesting that ZmMRP4 could have a direct or indirect role in anthocyanin transport [62].

To overcome the negative pleiotropic effects present in maize and rice, *lpa* mutants affected in *ZmMRP4* and *OsMRP5* genes, respectively, seed-specific silencing of both *MRP* genes was undertaken [24,42]. Transgenic lines expressing an antisense sequence for a fragment of the cDNA for the ZmMRP4 transporter under the control of the embryo-specific *Ole16* and *Glb* promoters produced *lpa*, high P_i_ grains that germinated normally and did not have any significant reduction in grain dry weight, revealing the potential of this approach in maize nutritional quality improvement [24]. On the other hand, plants silenced in the *OsMRP5* gene through the artificial microRNA (amiRNA) technology, under the control of the *Ole18* promoter, active in the embryo and aleurone, produced *lpa* grains (PA reduced by 35.8–71.9% with increased levels of P_i_ of up to 7.5 times). Although no consistent significant differences of plant height or number of tillers per plant were observed, significantly lower grain weights (up to 17.8% reduction) and reduced seed germination were observed, suggesting that this strategy is not successful for practical application in rice breeding. The different results obtained in maize and rice may depend on the different promoters used, with the rice ones also being active in aleurone and endosperm beyond the embryo [42]. A similar approach was also used in hexaploid wheat, where the three copies of the *TaABCC13* gene, previously shown to encode a protein able to transport cadmium [73] were silenced through RNA-interference (RNAi). In transgenic lines, a reduction in PA content of 34–22% was observed. Moreover, these lines were characterized by reduced grain filling, reduced numbers of spikelets, reduced kernel viability, delayed germination, early emergence of lateral roots, and defects in metal uptake and development of lateral roots in the presence of cadmium stress, compared to non-transgenic lines. These data show that TaABCC13 is important for several other aspects of growth as well as for grain nutritional quality and for root development and detoxification of heavy metals [33].

Mutations in PA-MRP transporters have also been reported in soybean and common bean, two of the most relevant legume crops worldwide [43,44]. Following EMS mutagenesis of the soybean breeding line *CX1515-4*, the two independent *M153* and *M766* mutant lines were isolated, with the *M153* line displaying a stronger PA reduction compared to the *M766* one (80% vs. 76.3%, respectively) [43]. However, the content of PA drops to 94% of that of the parental line when the double mutant is produced [31,43,74,75]. Although at the beginning it was hypothesized that a mutation in the *MIPS* gene could be responsible for the *lpa* phenotype of these lines [29,76], genetic and fine-mapping studies revealed that the trait was under the control of two loci, named *lpa1* and *lpa2* [74,77]. These contained independent but interactive recessive alleles coding for PA-MRP transporters, GmMRP3/GmABCC1, and GmMRP19/GmABCC2, respectively [30,31] (Table 1). It was shown that the *lpa1-a* allele (line M153) carries a nonsense mutation at R893, which results in a truncated protein [29,30], while in the case of the *lpa1-b* allele (M766 line) a single T > A SNP 7 bp upstream of the start of exon 10 was identified, which introduced an alternative splicing site producing five additional base pairs from the intron sequence and a frame shift starting at exon 10. Concerning the second locus, an R1039K change was identified in the *lpa2-a* allele (*M153* line), while in the *lpa2-b* allele (*M766* line) a single base change at position 1039 causes a premature termination [31].

A number of agronomic analyses have been performed on the soybean breeding line *CX1834-1-6* (derived from the mutant lines *M153*), and in different studies, a reduction in seedling emergence (about 22–30% less than wt) has been reported [75,78,79,80]. In particular, Anderson et al. (2008) demonstrated that the environment of reproduction of the *lpa* plants has important implications for seedlings’ field emergence. In fact, *lpa* seeds harvested in Puerto Rico (tropical environment) displayed decreased germination, compared to those harvested in Iowa (temperate environment). However, genetic improvement through advanced backcrossing was successful and *lpa* lines with normal seedling emergence were obtained [79].

In common bean, two *lpa* mutants in the PA-MRP transporter have been isolated in two different backgrounds [35,44]. In the *lpa1* mutant, a highly conserved Glu changed to Lys at position 1155, in the transmembrane domain TMD2, while in the *lpa1*^2^ mutant a single base pair change in the first exon caused a non-sense mutation (R500Stop) leading to a truncated protein. Reduction of PA accumulation was about 90% and 75% compared with the wt parent, for the *lpa1* and *lpa1*^2^ mutants, respectively, suggesting a highly critical functional role of the conserved Glu_1155_ residue. In the *lpa1* mutant, it has also been demonstrated that PA accumulation is accompanied by a decrease of raffinose-containing sugars by 25% and *myo*-inositol by 30% [32,44], thus indicating metabolic rearrangements of derived pathways. Despite the strong PA reduction in the seed, the different bean *lpa1* mutant lines showed that seedling emergence, seed yield, and plant growth were not statistically different from those of wt and parental genotypes [81]. Furthermore, germination of *lpa1* seeds in stressful conditions: By the accelerated aging test (AAT) and the stress integrated germination test (SIGT) showed that there was equal (SIGT) or even better (AAT) germination performance of *lpa1* seeds compared to the wt ones [44]. The finding that in common bean a second gene, *PvMRP2*, paralog of *PvMRP1*, is present, indicates that most likely it is able to complement the absence of a functional PvMRP1 in tissues and organs other than the seed. Another interesting feature of the *lpa1* mutant is that its seeds when germinated in the presence of ABA were hypersensitive to the presence of this phytohormone [26] a result contrasting with the finding of Klein and coworkers who reported that the Arabidopsis *mrp5* mutant has reduced sensitivity to ABA during germination [26]. Since the sensitivity of seed germination to ABA has been reported to correlate negatively to seed *myo*-inositol content [32,50,82,83], it is possible that *myo*-inositol levels are not reduced or may even be increased in the Arabidopsis *mrp5* mutant.

## 3. SULTR3.3 and SULTR3.4 Transporters Involved in PA Metabolism

Two *lpa* mutants isolated in barley and rice are affected in *HvST* and *OsSULTR3;3* genes, respectively [19,28,45,84], coding for two putative sulfate transporters, belonging to the SULTR3;3 class [18]. Recently, another rice *lpa* mutant, affected in the OsSULTR3;4 putative sulfate transporter, also called SULTR-like Phosphorus Distribution Transporter (SPDT), was isolated [20].

Here, we present an in silico analysis of *SULTR3;3* and *SULTR3;4* genes, including the ones already described (Table 1) and also putative *SULTR3;3* and *SULTR3;4* orthologs from other cereal and legume crops for which interest in the isolation of *lpa* mutants is considered an important challenge, such as maize, barley, common bean and soybean (Table 2). A phylogenetic tree with all the SULTR3;3 and SULTR3;4 proteins of cereals and legumes analyzed in the present work is shown in Figure S3.

### 3.1. SULTR3;3

**Figure 3:**
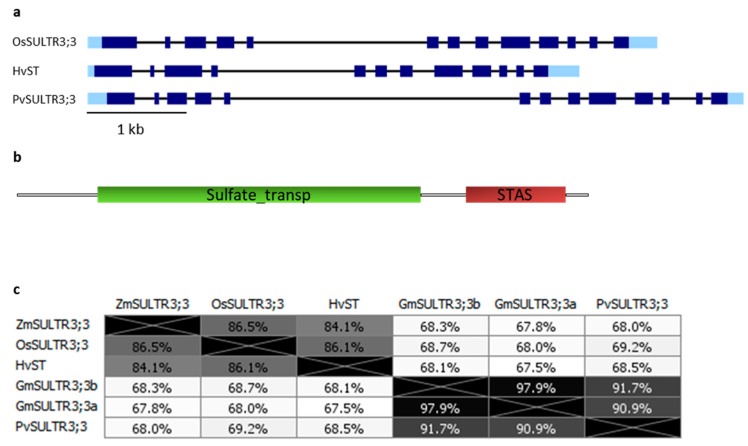
(**a**) Gene structure of *OsSULTR3;3, HvST* and putative *PvSULTR3;3* genes. Light and dark blue rectangles represent UTRs and coding exons, respectively, the black bars correspond to introns. Table 1. a legend. (**b**) Predicted domains of the SULTR3;3 protein. The sulfate transporter and the anti-sigma factor antagonist (STAS) domains are represented in red and green, respectively. Picture reproduced from [84]. (**c**) Distances between SULTR3;3 proteins, expressed as the percentage of identity. Phylogenies were constructed as described in Figure 1c.

In all analyzed species, one putative ortholog belonging to the SULTR3;3 group was found by BLAST analysis of HvST or OsSULTR3;3 against the different genomes, except for soybean, in which two different genes have been identified (Table 2, Figures S3 and S4). Indeed, this is not unexpected, as soybean underwent an ancient event of genome duplication [85]. The SULTR3;3 gene structure is quite conserved among species and consists of 13 exons in the majority of the genes, with the exceptions of barley and maize with only 12 exons. All genomic sequences are characterized by the presence of a long fourth or fifth intron, as reported in Figure 3a, where the structure of the characterized OsSULTR3;3 and HvST and of PvSULTR3;3 is shown as an example.

Predicted domains of SULTR3;3 proteins are represented in Figure 3b and correspond to a sulfate transporter domain and an anti-sigma factor antagonist (STAS) domain, as previously reported [84]. Protein length varies from 647 aa of PvSULTR3;3 to 661 aa of OsSULTR3;3 (Figure S4).

Protein identity is generally very high among different species, ranging from 84.1% to 86.5% among the considered cereals and from 90.9% to 91.7% among legumes, and at 97.9% in the two paralogs of soybean, as shown in the Figure 3c diagram.

In the case of the OsSULTR3;3 gene detailed expression analysis was reported: Transgenic lines harboring the promoter of this gene fused to the GUS reporter gene revealed that a strong GUS activity was present in vascular bundles of shoots, leaves, flowers, and grains, where it was mainly detected in the scutellum. Moreover, the subcellular localization was defined to be in the endoplasmic reticulum [84]. Interestingly, both GmSULTR3;3a and GmSULTR3;3b are expressed in leaves and flowers, while only GmSULTR3;3a was expressed in the seed, with an increasing expression during seed development with a peak at 35 DAF (data not shown, in silico analysis performed using the soybean eFP Browser [47]).

The exact function of this family of proteins is still unknown and in the case of OsSULTR3;3, which was the only one analyzed in detail, no activity was revealed for the transport of phosphate, sulfate, inositol or inositol 1,4,5 triphosphate by heterologous expression in either yeast or Xenopus oocytes [84].

### 3.2. SULTR3;4

**Figure 4:**
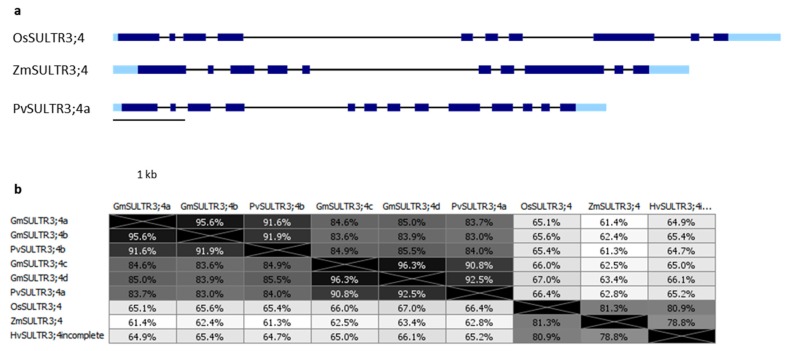
(**a**) Gene structure of *OsSULTR3;4* and putative *ZmSULTR3;4* and *PvSULTR3;4a* genes. Light and dark blue rectangles represent UTRs and coding exons, respectively, the black bars correspond to introns. The gene structure was obtained as described in Figure 1a legend. (**b**) Distances between SULTR3;4 proteins, expressed as the percentage of identity. Phylogenies were constructed as described in Figure 1c.

In the case of the SULTR3;4 group of transporters a similar situation to the one previously described for MRP proteins is present: In cereals, only one protein for each species can be found by BLAST analysis of OsSULTR3;4 against the different genomes, while in legumes, two or four paralogous proteins are present in common bean and soybean, respectively (Table 2 and Figures S3 and S5). The gene structure differs between cereals with 10 exons (the barley sequence present in the Phytozome database is incomplete with only eight exons) and legumes with 13 exons and also in this case, the fourth (the fifth in maize) intron is quite long. In Figure 4a the structures of the characterized OsSULTR3;4 and of putative ZmSULTR3;4 and PvSULTR3;4a genes are given as examples.

Predicted domains are the same as those already described for SULTR3;3, represented in Figure 3b. Protein length varies from 648 aa of the soybean protein to 670 aa of the rice one (Figure S5).

Also for SULTR3;4 proteins, identity is quite high ranging from 78.8% to 81.3% among cereals, from 83% to 91.9% among legumes and from 61.3% to 67% between cereals and legumes, as shown in the diagram in Figure 4b.

Analysis of the phylogenetic tree (Figure S3) clearly shows a separation between monocotyledons and dicotyledons. Furthermore, in the two legume species, the gene is duplicated, with soybean carrying four genes arising from an ancient event of genome duplication [85].

qRT-PCR expression analysis of OsSULTR3;4 gene revealed that during grain filling it was mainly expressed in node I, a very important hub for mineral distribution to upper node and panicle in Poaceae [86]. Moreover, immunostaining against GFP in lines harboring OsSULTR3;4 promoter fused to GFP, showed the highest staining in the xylem region of both enlarged- and diffuse-vascular bundles of the basal node and in node I, as well as in the parenchyma tissues between them, but not in the phloem region [20]. The activity of OsSULTR3;4 as an influx plasma-membrane localized H+/P_i_ symporter was shown in proteoliposomes as well as in Xenopus oocytes. Particularly, it was found that OsSULTR3;4 is involved in the intervascular transfer of P at the nodes, unloading P from xylem towards phloem [20].

### 3.3. lpa Mutants in SULTR Transporters

The first mutants affected in SULTR3;3 and SULTR3;4 genes were described in *Arabidopsis thaliana*. They have been characterized for phenotypic alterations related to sulfate translocation between seed compartments [87]. Moreover, using the quintuple mutant defective in all SULTR3 subfamily members, it was recently shown that they have functional redundancy in chloroplast sulfate uptake and consequent influence on Cys, glutathione, and ABA biosynthesis, with the resulting growth retardation and altered stress responses in the multiple mutants [88]. Otherwise, no evidence of the involvement of these Arabidopsis genes in PA metabolism has been reported so far, with the only exception of AtSULTR3;4 for which contrasting results have been reported. In fact, very recently, Ding and co-workers [89] demonstrated that AtSULTR3;4/SPDT functions as a high-affinity P_i_ transporter, being able to mediate P_i_ uptake when injected in the Xenopus oocyte. Furthermore, it has been shown to localize to the plasma membrane, while Chen et al. reported a chloroplast localization [89]. On the other hand, these data are in agreement with those reported below on mutations affecting the HvST, OsSULTR3;3 and OsSULTR3;4 genes which confer grain *lpa* phenotype and in which the relevant proteins are localized in the endoplasmic reticulum and plasma membranes, respectively [19,20,84].

#### 3.3.1. Mutants Affected in the *SULTR3;3* Genes

In the case of *HvST* a nonsense mutation (*M422*) in the last exon of the barley *lpa1-1* gene was isolated from a sodium azide mutagenized population [19], and in the case of *OsSULTR3;3* the two different *Os-lpa-Z9B-1* and *Os-lpa-MH86-1* mutations were a 6 bp deletion in the first exon and a 1 bp deletion in the 12th exon, identified through screening of a gamma-ray irradiation mutagenized population [28,45,84]. These barley and rice mutants exhibit a decrease in phytic acid-P like other *lpa* mutants, but also a decrease in total P in the seed (about 15% in barley mutant and 27.5–18.9% in rice mutants) [19,84,90], differently from *lpa* mutants affected in biosynthetic or *MRP*-transporter genes. Particularly, an endosperm-specific total P reduction [90] was reported that is not due to a reduction in the uptake of P in the maternal plant, suggesting that HvST functions as a seed-specific or filial determinant of barley endosperm total P. Moreover, OsSULTR3;3 disruption dramatically alters the grain metabolite profile. In fact, an increase was observed in the concentration of sugars involved in the close biosynthetic pathway leading to PA, sugar alcohols, free fatty acids, organic acids, biogenic amine GABA, serine, and lysine. However, the concentration of cysteine was decreased [84]. These traits were also stably maintained in the homozygous *lpa* progeny of generations F4 to F7 of crosses between the original *Os-lpaMH86-1* mutant with a commercial rice cultivar [91]. In addition, the metabolic profiles of the *lpa* progenies were strongly influenced by the lipid profiles of the wild type cultivar used as the crossing parent [92].

The OsSULTR3;3 mutants also show a significant increase in seed total sulfur and in sulfate concentration in embryo and pericarp/aleuronic layers. The mutations also increase root and leaf P and P_i_ concentrations and decrease root and leaf sulfate concentration in comparison to their corresponding wild type parents. Moreover, the analysis performed on developing seeds of the *MH86* mutant showed that the expression of genes coding for the last steps of PA biosynthesis was altered: Generally, an up-regulation was shown, and the expression of genes for sulfur metabolism and sulfate transport was different in the mutant compared to the corresponding wild type. However, the most dramatic effects on gene expression concern several genes involved in P signaling and homeostasis [84]. A redistribution of P_i_ in endosperm and a reduction of lysophospholipid content were also observed in the rice mutant [93].

As previously mentioned, the role of the SULTR3;3 transporter is not clear, as, when expressed in heterologous systems, such as yeast or Xenopus oocytes, it is unable to transport either sulfate, or phosphate, or PA precursors [84]. However, it cannot be excluded that in plant systems OsSULTR3;3 may transport these molecules as well as PA. In plant cells OsSULTR3;3 is ER-localized. Previous studies have suggested that the final steps of PA synthesis (from InsP3 to InsP6) take place in the ER [94]. Zhao and collaborators suggested that OsSULTR3;3 may have a specific role in the existing cross-talk between sulfate and phosphate homeostasis and/or signaling, as it has effects on phosphate as well as on sulfate concentrations in both vegetative tissues and grain [84].

Unfortunately, from an agronomic point of view, these mutants show some negative pleiotropic effects. In the rice mutant, grain weight reduction and yield per plant reductions have been shown [45]. In barley, only in rain-fed locations and not in irrigated ones, the mutation is associated with reduced test weight and percentage of plump kernels [95].

Interestingly, the mutant barley straws, although not showing significant differences in terms of fiber composition, compared to the wild type, after an acidic pre-treatment, showed increased fiber hydrolysibility, thus representing a promising material for cellulosic ethanol production [96].

#### 3.3.2. The *spdt* Mutants

The rice *spdt* mutants, affected in the *OsSULTR3;4/SPDT* gene are retrotransposon *Tos-17* insertion lines (the transposon is in the fourth exon in *spdt1* and in the eighth exon in *spdt-2* and *spdt-3*). The analysis of these mutants, grown under field conditions, revealed that the distribution of P in different organs was greatly altered, with a reduction by 20% of P concentration and P content in the seeds, without a significant penalty on grain yield, and a comparable 20% increase of P in the straw. Moreover, in the mutant seeds, a reduction in the concentration of PA by 25–32% was observed, compared to the wild type. However, neither the seed germination rate nor the early growth was affected by the reduced phytate content. P in the grain comes from re-translocation from old leaves or from node-based distribution of P newly taken up after the flowering stage. The reported results indicate that SPDT, localized in the nodes, especially in the uppermost node I, functions as a switch for P distribution to the grains. Indeed, another meaning of the acronym SPDT, used by Yamaji et al. (2017), is “single-pole, double throw”, corresponding to a type of two-way electrical switch.

As knockout of *SPDT* resulted in a 20% reduction of total P and about 30% of PA in the grain without an obvious penalty of grain yield, and in increased P in the straw, the use of these mutants may present some advantages: As straw will be returned to the field after harvest, less P will be removed from the field, reducing the requirement for P fertilizer input. Their *lpa* phenotype may increase mineral bioavailability and lower the risk of eutrophication of waterways [20]. Very recently, the *atsultr3;4* mutant of Arabidopsis has been characterized and demonstrated to be a high-affinity P_i_ transporter that mediates xylem to phloem transfer of phosphate. In particular, it has been shown that, like the *OsSULTR3;4/SPDT* mutant, *atsultr3;4* seeds accumulate less P (about 15%) than the wt ones. This decrease is accompanied by a P increase in the shoot, indicating a role of AtSULTR3;4/SPDT in mediating P allocation to the seeds [89].

## 4. OsPht1;4 Phosphate Transporter

The OsPht1;4 (or OsPT4, corresponding to LOC_Os04g10750) phosphate transporter, belonging to the Pht1 family, was described as influencing grain PA content, as the corresponding mutant produces *lpa* grains [21]. As the identification of putative orthologs of this protein in other species is not so obvious, due to the high number of Pht1 genes and to their sequence similarity, (in rice there are 26 [97]), in the present review we limit our consideration to OsPT4.

**Figure 5:**
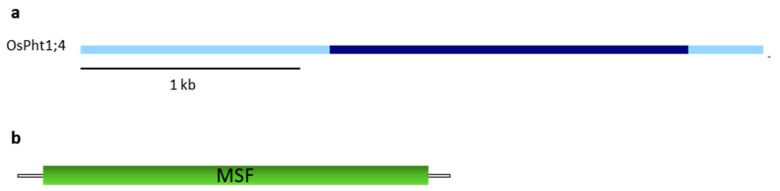
(**a**) Gene structure of OsPHT1;4. Light and dark blue rectangles represent UTRs and coding exons, respectively, the black bars correspond to introns. The gene structure was obtained as described in Figure 1a legend. (**b**) Predicted domain of the OsPHT1;4 protein by PFAM [98] software. The major facilitator superfamily (MFS) domain is represented.

The genomic sequence is characterized by the presence of a single exon (Figure 5a) and the protein, 538 aa long, by a major facilitator superfamily domain, characteristic of different transporters, including phosphate transporters (Figure 5b).

The *OsPT4* gene is mainly expressed in roots, flag leaves and embryos, and its expression is increased in response to prolonged P starvation conditions in shoots and roots, where the signal is specifically localized to the exodermis. The protein is localized to the plasma membrane, as shown in the protoplast system and it is a functional P_i_ influx transporter, able to complement a yeast mutant defective in P_i_ uptake and to facilitate the increased accumulation of P_i_ in Xenopus oocytes.

### Ospt4 Mutants, OsPT4 RNAi and Overexpression Lines

The OsPT4 functional characterization was performed using transposon insertional mutants, knockdown lines harboring the *OsPT4-RNAi* construct and overexpression lines. P_i_ and total P concentration is strongly reduced in mutant lines, attenuated in RNAi lines and increased in overexpression lines, in roots as well as in shoots. Moreover, a dramatic reduction in P_i_ uptake in mutants, a small reduction in RNAi lines, and an increase in overexpression lines were observed [21,22]. There was altered expression of genes that regulate Pi absorption and homeostasis, such as *OsPHO2*, *OsPHR2*, and *OsSPX1*. A detailed analysis of the grains revealed a decrease in total P concentration in the embryo and in one line also in the endosperm, attenuated and increased effects in RNAi lines and overexpression lines, respectively. Moreover, a decrease of 32–22% in PA concentration was observed in *ospt4-1* and RNAi lines’ grains and an increase of 10% in overexpression lines. The alteration in mutant and RNAi embryos correlates with a reduction in the transcript levels of *OsRINO* (coding for *myo*-inositol 3-phosphate synthase—MIPS) and of *OsIPK1* (coding for 1,3,5,6-pentakisphosphate 2-kinase), coding for enzymes catalyzing the first and the last step of the PA biosynthetic pathway, respectively. Both genes’ transcript levels were also significantly reduced in the endosperm of mutant grains and only partially reduced in RNAi lines [21]. From all these data, it is clear that OsPT4 has an important role in acquisition and mobilization of P_i_ and also during embryogenesis and seed development, so it is a good candidate to improve P efficiency, although alterations in panicle robustness, grain-setting rates, grain weight, grain yield per plant and seed germination registered in *ospt4* and *OsPt4* RNAi lines need breeding actions to ensure acceptable agronomic performance and avoid yield penalties.

## 5. Conclusions

**Figure 6:**
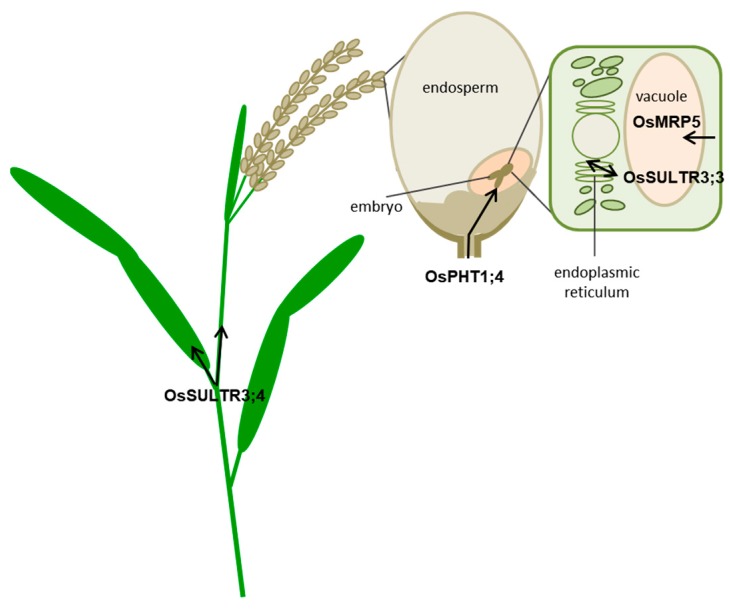
Rice transporters identified to modulate PA homeostasis. Modified from [99].

Strategies for controlling the accumulation of specific metabolites are commonly based on switching off structural or regulatory genes of the biosynthetic pathway. However, in order to avoid or reduce downstream effects on derived pathways, a different approach is to interfere with compound transport to the site of accumulation (organ, cell type, subcellular compartment). In this review we reported data showing how PA reduction can be achieved with mutations in different types of transporters that control PA transport to the vacuole (MRP), or by modifying P_i_ availability for PA synthesis through mutations in transporters involved in Pi loading and organ/intracellular distribution (SULTR) or by P_i_ acquisition and mobilization during seed development (PHT1;4) (Figure 6).

These types of *lpa* mutants are potentially advantageous over other *lpa* mutants in structural biosynthetic genes to achieve a triple goal: (i) Increased bioavailability of mineral cations, which are no longer chelated by PA, (ii) less PA is excreted into the environment with manures, and hence there is reduced impact on water eutrophication, (iii) increased P use efficiency as the seeds are loaded with less P which remains in the straw and may potentially contribute to reducing the demand for P fertilizers, hence increasing crop sustainability [99].

From an agronomic point of view, mutants in *MRP* and *PHT1;4* genes show compromised yields, seed setting, seed germination, and/or seedling growth, etc., so that they are not very attractive for breeders [21,22,37]. However, good field performance has been demonstrated for *MRP* mutants of common bean, which is due to the presence of duplicated *MRP* gene(s) able to complement the mutated seed-specific copy [32]. So far, *sultr3;3* and *sultr3;4/spdt* mutants, besides Arabidopsis, have been isolated and described only in rice and barley. Interestingly, these mutants do not display negative pleiotropic effects as observed for the other transporters described above, hence they potentially represent a valuable tool to simultaneously achieve seed biofortification and a more sustainable crop while reducing the environmental impact of the crop cultivation. It would be very interesting to verify the role and function of *sultr3;3* and *sultr3;4/spdt* genes in legume crops as well, since contrasting data on function and subcellular localization have been reported for Arabidopsis mutants and a possible species-specific and/or development-specific behavior has been proposed [100].

Finally, the finding that both phosphate and putative sulfate transporters produce similar *lpa* phenotypes suggests the “existence of a multilevel coordination in the regulation of the two ions in which currently unidentified key elements are actively cross-talking between the two signaling pathways” [101,102]. The availability of such mutants from different crops may help towards understanding this cross-talk and identifying new players.

## Figures and Tables

**Table 1:** Described cereal and legume transporters involved in PA accumulation and corresponding mutations affecting PA seed/grain content. EMS stands for ethyl methanesulfonate. * Indicates incomplete sequence.

Class of Transporters	Species	Gene	Phytozome/Genbank/Ensembl Accession Number	Origin of Mutation	Mutation	Reference
MRP	*Zea mays*	*ZmMRP4/ZmABCC4*	EF586878	EMS	*lpa1-1*	[37]
					*lpa1-241*	[38]
					*lpa1-7*	[39]
				T-DNA insertion	*lpa1-mum1*	[24]
				Embryo specific:RNAi	*Ole::MRP4 Glb::MRP4*	[24]
	*Oryza sativa*	*OsMRP5/OsABCC13*	LOC_Os03g04920	γ rays + sodium azide	*Os-lpa-XS110-2*	[40]
					*Os-lpa-XS110-3*	[41]
				T-DNA insertion	*4A-02500*	[41]
				Embryo specific amiRNA	*Ami-MRP5*	[42]
	*Triticum aestivum*	*TaABCC13-4B*	TraesCS4B02G343800	Constitutive RNAi	*TaABCC13 RNAi*	[33]
		*TaABCC13-4D*	TraesCS4D02G339000			
		TaABCC13-5A*	TraesCS5A02G512500			
	*Glycine max*	*GmMRP3/GmABCC1*	Glyma.03G167800	EMS	*CX1834*	[29,30,31,43]
		*GmMRP19/GmABCC2*	Glyma.19G169000			
		*GmMRP13/GmABCC3*	Glyma.13G127500	no reported mutant	no reported mutant	[32]
	*Phaseolus vulgaris*	*PvMRP1/PvABCC1*	Phvul.001G165500	EMS	*lpa1*	[32,44]
					*lpa1^2^*	[35]
		*PvMRP2/PvABCC2*	Phvul.007G153800	no reported mutant	no reported mutant	[32]
SULTR	*Oryza sativa*	*OsSULTR3;3*	LOC_Os04g55800	γ rays	*Oslpa-MH86-1* *Os-lpa-Z9B-1*	[28] [19]
		*OsSULTR3;4*	LOC_Os06g05160	retrotransposon *Tos-17* insertion	*spdt-1, spdt-2, spdt-3*	[20]
	*Hordeum vulgare*	*Hvst*	HORVU2Hr1G113050	sodium azide	*lpa1-1(M422)*	[45] [19]
Pht	*Oryza sativa*	*OsPht1;4*	LOC_Os04g10750	retrotransposon *Tos-17* insertion	*ospt4-1 (NE1260) ospt4-2 (SHIP_ZSF6267)* RNAi	[21] [22]

**Table 2:** Putative orthologous genes of *OsSULTR3;3/HvSULTR3;3* and *OsSULTR3;4*, identified in maize, common bean, and soybean by in silico analysis.

SULTR Group.	Species	Gene Name	Phytozome Accession Number
SULTR3;3	*Zea mays*	*ZmSULTR3;3*	GRMZM2G395114
	*Phaseolus vulgaris*	*PvSULTR3;3*	Phvul.002G095300
	*Glycine max*	*GmSULTR3;3a*	Glyma.20G017100
		*GmSULTR3;3b*	Glyma.07G218900
SULTR3;4	*Zea mays*	*ZmSULTR3;4*	GRMZM2G444801
	*Phaseolus vulgaris*	*PvSULTR3;4a*	Phvul.005G171800
		*PvSULTR3;4b*	Phvul.010G151000
	*Glycine max*	*GmSULTR3;4a*	Glyma.07G006500
		*GmSULTR3;4b*	Glyma.08G207100
		*GmSULTR3;4c*	Glyma.13G360000
		*GmSULTR3;4d*	Glyma.15G014000

## Supplementary Materials

The following are available online at https://www.mdpi.com/2223-7747/9/1/69/s1. Figure S1. PA-MRP proteins alignment. See Table 1 for the correspondence with genes accession numbers. The Clustal W alignment (cost matrix Blosum, gap open cost 10, gap extend cost 0.1) of Geneious 11.0.2 software was used. Figure S2. Phylogenetic tree of characterized crop PA-MRP proteins, listed in Table 1. Phylogenies were constructed with the Geneious Tree Builder tool, using the Jukes-Cantor distance model, Neighbor-Joining tree build method. Figure S3. Phylogenetic tree of SULTR3;3 and SULTR3;4 proteins, listed in Table 2. Phylogenies were constructed as described in Figure S2. Figure S4. SULTR3;3 proteins alignment. See Table 1 and Table 2 for the correspondence with genes accession numbers. The method used is described in Figure S1 legend. Figure S5. SULTR3;4 proteins alignment. See Table 1 and Table 2 for the correspondence with genes accession numbers. The method used is described in Figure S1 legend.
