# Supplementary material for: Phytic Acid and Transporters: What Can We Learn from low phytic acid Mutants?
Source: Plants (Basel). 2020 Jan 5;9(1):69. doi: 10.3390/plants9010069 (PMC7020491; doi:10.3390/plants9010069)
Supplement: Supplementary file 1 [file plants-09-00069-s001.zip › Figure S1.pdf]

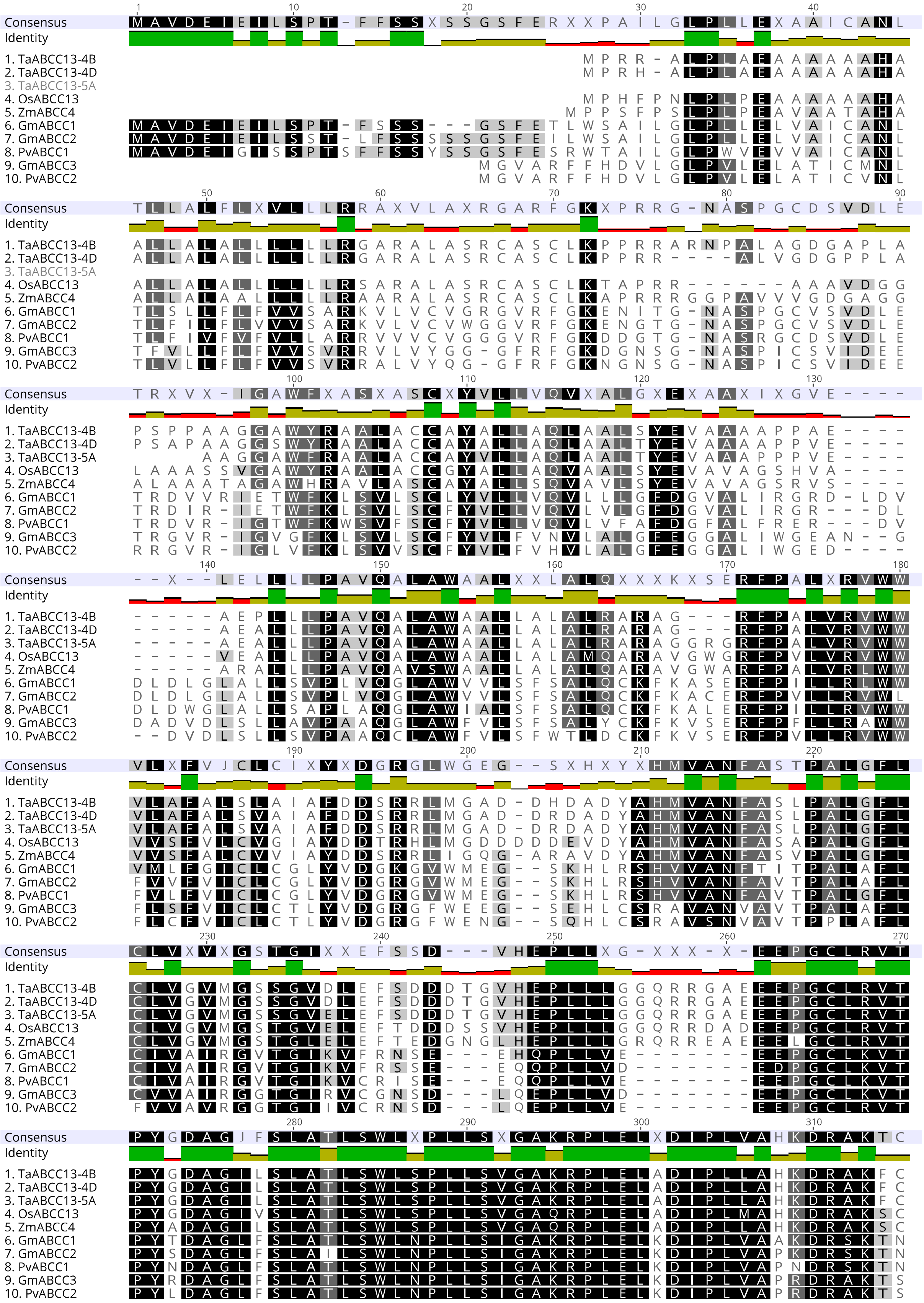
Consensus
Identity
1. TaABCC13-4B
2. TaABCC13-4D
3. TaABCC13-5A
4. OsABCC13
5. ZmABCC4
6. GmABCC1
7. GmABCC2
8. PvABCC1
9. GmABCC3
10. PvABCC2

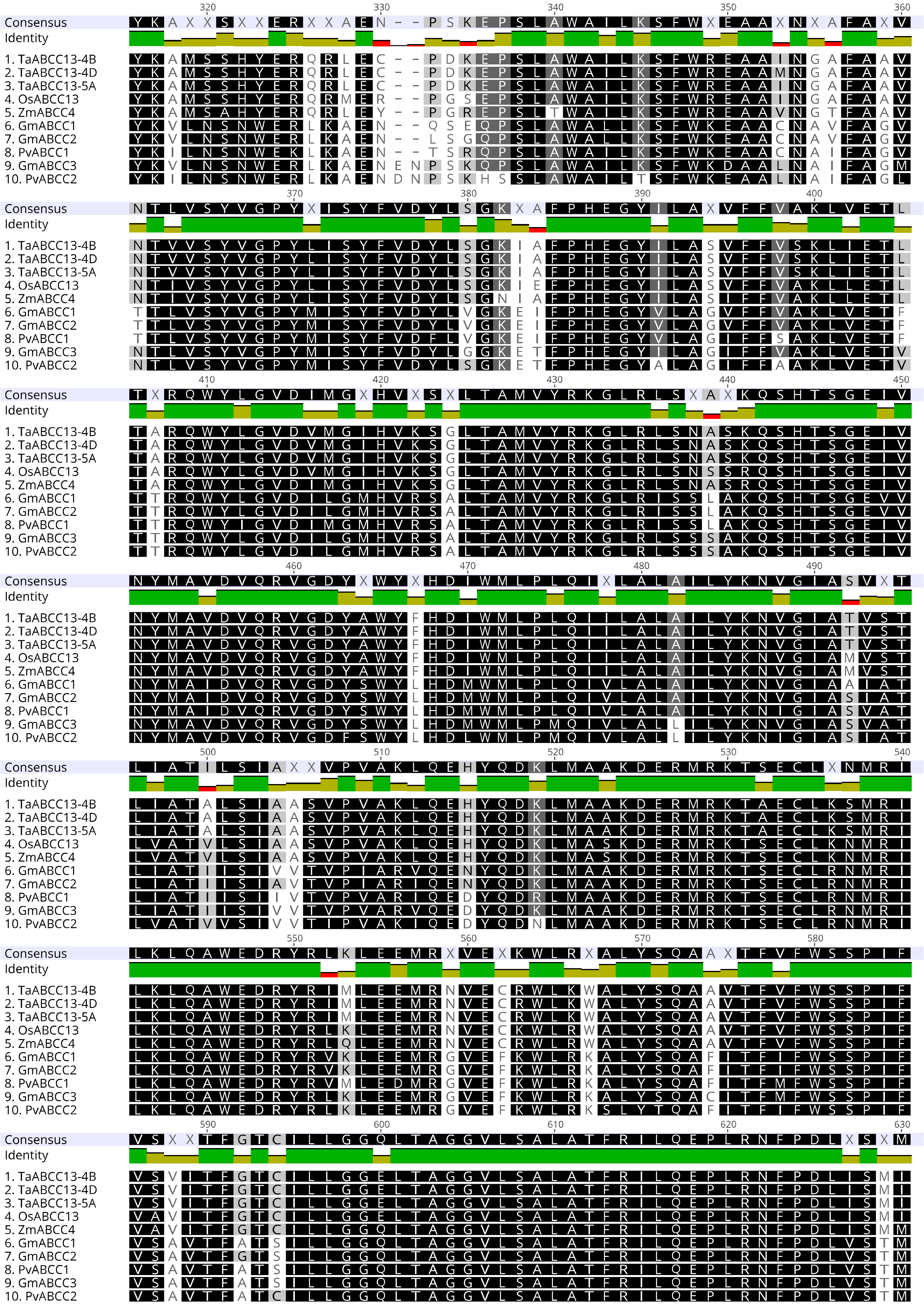
Consensus
Identity
1. TaABCC13-4B
2. TaABCC13-4D
3. TaABCC13-5A
4. OsABCC13
5. ZmABCC4
6. GmABCC1
7. GmABCC2
8. PvABCC1
9. GmABCC3
10. PvABCC2

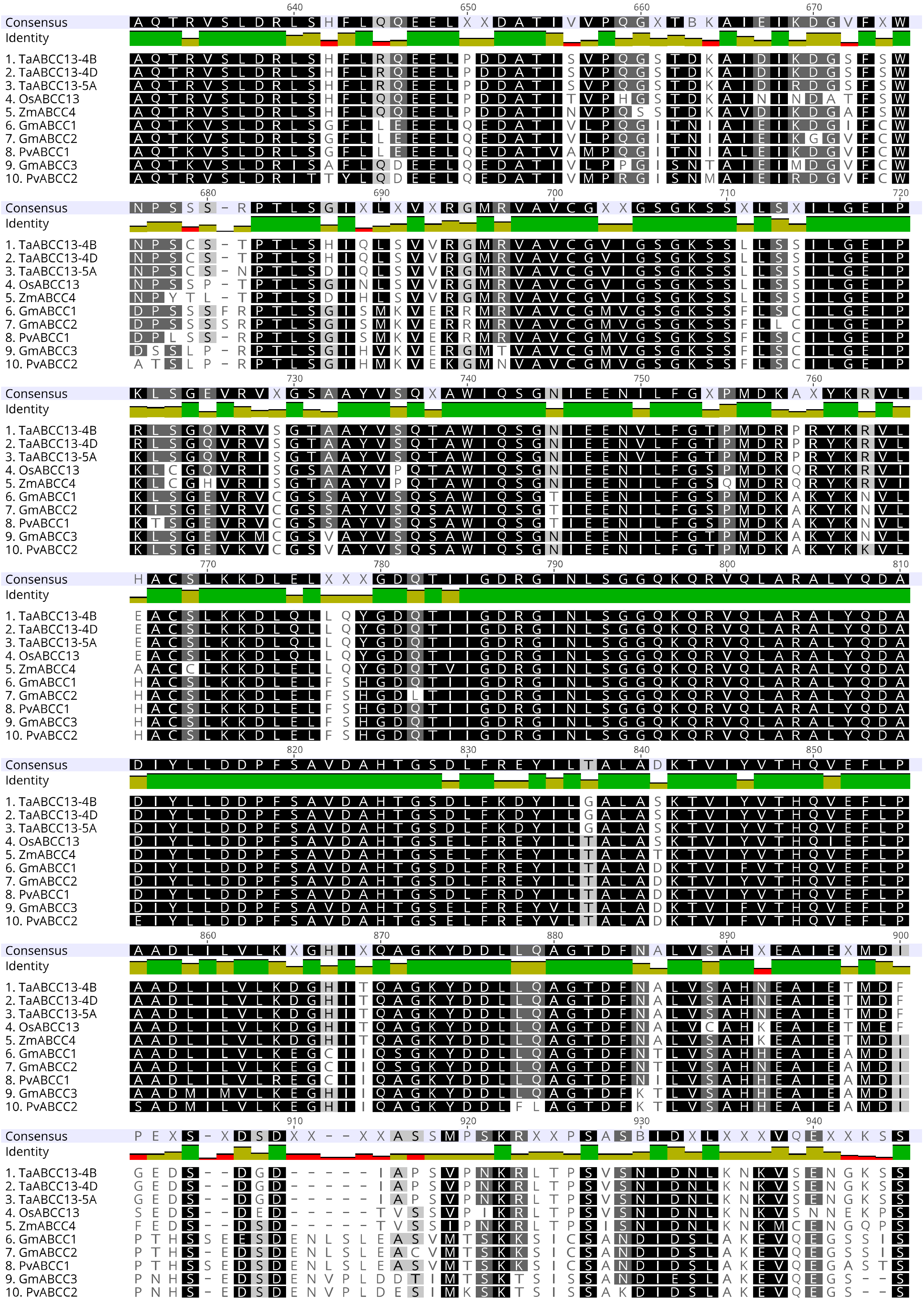
Consensus
Identity
1. TaABCC13-4B
2. TaABCC13-4D
3. TaABCC13-5A
4. OsABCC13
5. ZmABCC4
6. GmABCC1
7. GmABCC2
8. PvABCC1
9. GmABCC3
10. PvABCC2

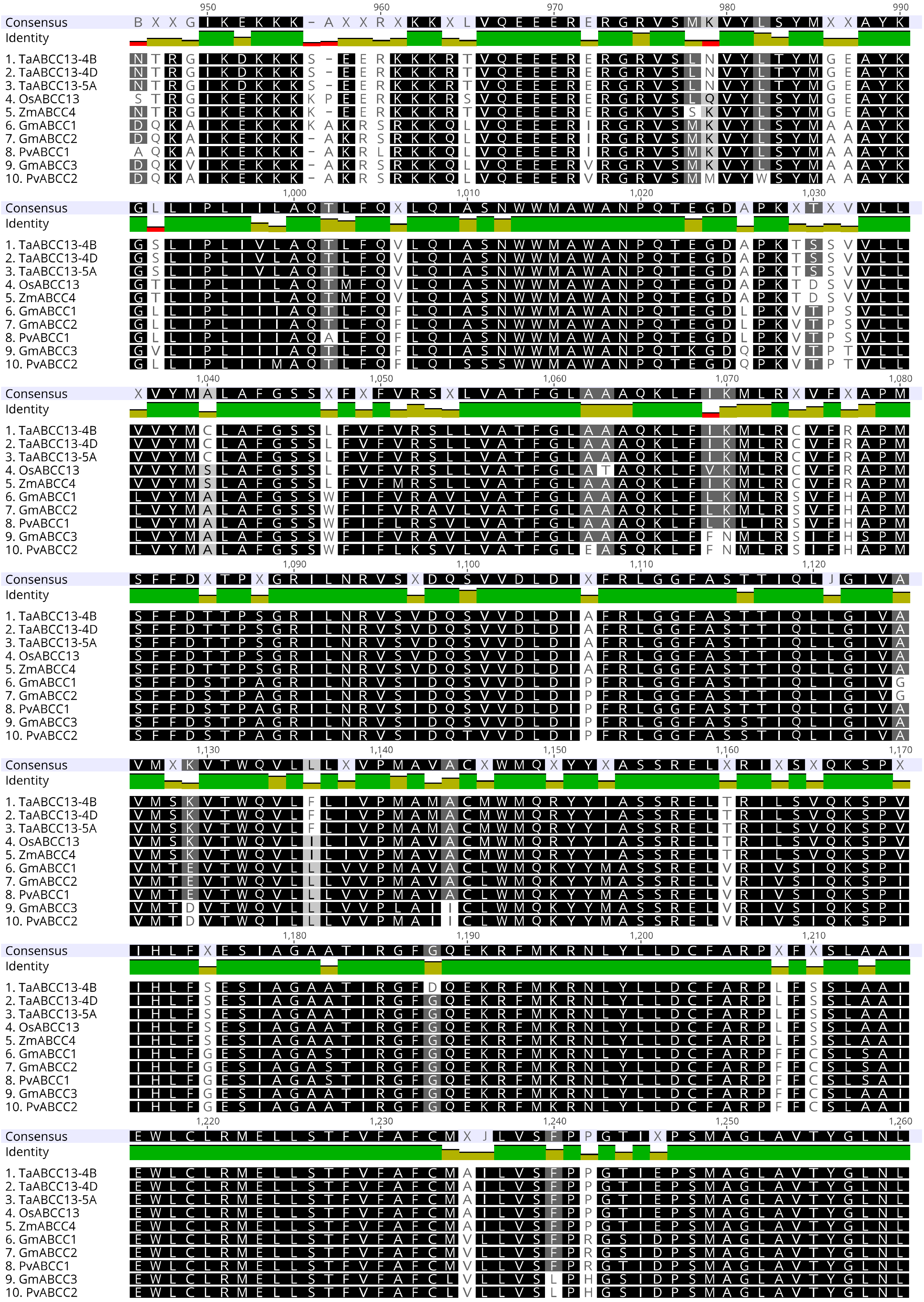
950 960 970 980 990
Consensus BXXGIKEKKK-AXXRXKKXLVQEEEREREGRVSMKVYLSYMXXAYK
Identity
1. TaABCC13-4B NTRGIKDKKKS-EERKKKRTVQEEEREREGRVSLNVYLTYMGEAYK
2. TaABCC13-4D NTRGIKDKKKS-EERKKKRTVQEEEREREGRVSLNVYLTYMGEAYK
3. TaABCC13-5A NTRGIKDKKKS-EERKKKRTVQEEEREREGRVSLNVYLTYMGEAYK
4. OsABCC13 STRGIKEKKKKPEERKKKRSVQEEEREREGRVSLQVYLSYMGEAYK
5. ZmABCC4 NTRGIKEKKKK-EERKKKRTVQEEEREREGKVSSKVYLSYMGEAYK
6. GmABCC1 DQKAIKEKKKKAKRSRKKQLVQEEERIRGRVSMKVYLSYMAAAYK
7. GmABCC2 DQKAIKEKKK-AKRSRKKQLVQEEERIRGRVSMKVYLSYMAAAYK
8. PvABCC1 AQKAIKEKKK-AKRLRKKQLVQEEERIRGRVSMKVYLSYMAAAYK
9. GmABCC3 DQKVIKEKKK-AKRSRKKQLVQEEERVRGRVSMKVYLSYMAAAYK
10. PvABCC2 DQKAIKEKKK-AKRSRKKQLVQEEERVRGRVSMMVYWSYMAAAYK
1,000 1,010 1,020 1,030
Consensus GLLIPLIILAQTLFQXLQIASNWWMAWANPQTEGDAPKXTXVVLL
Identity
1. TaABCC13-4B GSLIPLIVLAQTLFQVLQIASNWWMAWANPQTEGDAPKTSSVVLL
2. TaABCC13-4D GSLIPLIVLAQTLFQVLQIASNWWMAWANPQTEGDAPKTSSVVLL
3. TaABCC13-5A GSLIPLIVLAQTLFQVLQIASNWWMAWANPQTEGDAPKTSSVVLL
4. OsABCC13 GTLIPLIILAQTMFQVLQIASNWWMAWANPQTEGDAPKTDSVVLL
5. ZmABCC4 GTLIPLIILAQTMFQVLQIASNWWMAWANPQTEGDAPKTDSVVLL
6. GmABCC1 GLLIPLIIIAQTLFQFLQIASNWWMAWANPQTEGDLPKVTPSVLL
7. GmABCC2 GLLIPLIIIAQTLFQFLQIASNWWMAWANPQTEGDLPKVTPSVLL
8. PvABCC1 GLLIPLIIIAQALFQFLQIASNWWMAWANPQTEGDLPKVTPSVLL
9. GmABCC3 GVLIPLIIIAQTLFQFLQIASNWWMAWANPQTKGDQPKVTPTVLL
10. PvABCC2 GLLIPLIIMAQTLFQFLQISSSSWWMAWANPQTEGDQPKVTPTVLL
1,040 1,050 1,060 1,070 1,080
Consensus XVYMALAFGSSXFXFVRSXLVATFGLAAAQKLFIKMLRXVFXAPM
Identity
1. TaABCC13-4B VVYMCLAFGSSLFVFVRSLLVATFGLAAAQKLFIKMLRCVFRAPM
2. TaABCC13-4D VVYMCLAFGSSLFVFVRSLLVATFGLAAAQKLFIKMLRCVFRAPM
3. TaABCC13-5A VVYMCLAFGSSLFVFVRSLLVATFGLAAAQKLFIKMLRCVFRAPM
4. OsABCC13 VVYMSLAFGSSLFVFVRSLLVATFGLATAQKLFVKMLRCVFRAPM
5. ZmABCC4 VVYMSLAFGSSLFVFMRSLLVATFGLAAAQKLFIKMLRCVFRAPM
6. GmABCC1 LVYMALAFGSSWFIFVRAVLVATFGLAAAQKLFLKMLRSVFHAPM
7. GmABCC2 LVYMALAFGSSWFIFVRAVLVATFGLAAAQKLFLKMLRSVFHAPM
8. PvABCC1 LVYMALAFGSSWFIFLRSVLVATFGLAAAQKLFLKLIRSVFHAPM
9. GmABCC3 LVYMALAFGSSWFIFVRAVLVATFGLAAAQKLFFNMLRSIFHSPM
10. PvABCC2 LVYMALAFGSSWFIFLKSVLVATFGLEASQKLFFNMLRSIFHAPM
1,090 1,100 1,110 1,120
Consensus SFFDXTPXGRILNRVSXDQSVVDLDIXFRLGGFASTTIQLJGIVA
Identity
1. TaABCC13-4B SFFDTTPSGRILNRVSVDQSVVDLDIAFRLGGFASTTIQLLGIVA
2. TaABCC13-4D SFFDTTPSGRILNRVSVDQSVVDLDIAFRLGGFASTTIQLLGIVA
3. TaABCC13-5A SFFDTTPSGRILNRVSVDQSVVDLDIAFRLGGFASTTIQLLGIVA
4. OsABCC13 SFFDTTPSGRILNRVSVDQSVVDLDIAFRLGGFASTTIQLLGIVA
5. ZmABCC4 SFFDTTPSGRILNRVSVDQSVVDLDIAFRLGGFASTTIQLLGIVA
6. GmABCC1 SFFDSTPAGRILNRVSIDQSVVDLDIPFRLGGFASTTIQLIGIVG
7. GmABCC2 SFFDSTPAGRILNRVSIDQSVVDLDIPFRLGGFASTTIQLIGIVG
8. PvABCC1 SFFDSTPAGRILNRVSIDQSVVDLDIPFRLGGFASTTIQLIGIVA
9. GmABCC3 SFFDSTPAGRILNRVSIDQSVVDLDIPFRLGGFASSTIQLIGIVA
10. PvABCC2 SFFDSTPAGRILNRVSIDQTVVDLDIPFRLGGFASSTIQLIGIVA
1,130 1,140 1,150 1,160 1,170
Consensus VMXKVTWQVLLLXVPMAVACXWMQXYYXASSRELXRIXSXQKSPX
Identity
1. TaABCC13-4B VMSKVTWQVLFLIVPMAMACMWMQRYYIASSRELTRILSVQKSPV
2. TaABCC13-4D VMSKVTWQVLFLIVPMAMACMWMQRYYIASSRELTRILSVQKSPV
3. TaABCC13-5A VMSKVTWQVLFLIVPMAMACMWMQRYYIASSRELTRILSVQKSPV
4. OsABCC13 VMSKVTWQVLILIVPMAVACMWMQRYYIASSRELTRILSVQKSPV
5. ZmABCC4 VMSKVTWQVLILIVPMAVACMWMQRYYIASSRELTRILSVQKSPV
6. GmABCC1 VMTEVTWQVLLLVVPMAVACLWMQKYYMASSRELVRIVSIQKSPI
7. GmABCC2 VMTEVTWQVLLLVVPMAVACLWMQKYYMASSRELVRIVSIQKSPI
8. PvABCC1 VMTEVTWQVLLLVVPMAVACLWMQKYYMASSRELVRIVSIQKSPI
9. GmABCC3 VMTDVTWQVLLLVVPLAIICLWMQKYYMASSRELVRIVSIQKSPI
10. PvABCC2 VMTDVTWQILLLVVPMAIICLWMQKYYMASSRELVRIVSIQKSPI
1,180 1,190 1,200 1,210
Consensus IHLFXESIAGAATIRGFGQEKRFMKRNLYLLDCFARPXFXSLAAI
Identity
1. TaABCC13-4B IHLFSESIAGAATIRGFDQEKRFMKRNLYLLDCFARPLFSSLAAI
2. TaABCC13-4D IHLFSESIAGAATIRGFGQEKRFMKRNLYLLDCFARPLFSSLAAI
3. TaABCC13-5A IHLFSESIAGAATIRGFGQEKRFMKRNLYLLDCFARPLFSSLAAI
4. OsABCC13 IHLFSESIAGAATIRGFGQEKRFMKRNLYLLDCFARPLFSSLAAI
5. ZmABCC4 IHLFSESIAGAATIRGFGQEKRFMKRNLYLLDCFARPLFSSLAAI
6. GmABCC1 IHLFGESIAGASTIRGFGQEKRFMKRNLYLLDCFARPFFCSLSAI
7. GmABCC2 IHLFGESIAGASTIRGFGQEKRFMKRNLYLLDCFARPFFCSLSAI
8. PvABCC1 IHLFGESIAGASTIRGFGQEKRFMKRNLYLLDCFARPFFCSLSAI
9. GmABCC3 IHLFGESIAGASTIRGFGQEKRFMKRNLYLLDCFARPFFCSLAAI
10. PvABCC2 IHLFGESIAGAATIRGFGQEKRFMKRNLYLLDCFARPFFCSLAAI
1,220 1,230 1,240 1,250 1,260
Consensus EWLCLRMELLSTFVFAFCMXJLVSFPPGTIXPSMAGLAVTYGLNL
Identity
1. TaABCC13-4B EWLCLRMELLSTFVFAFCMAILVSFPPGTIEPSMAGLAVTYGLNL
2. TaABCC13-4D EWLCLRMELLSTFVFAFCMAILVSFPPGTIEPSMAGLAVTYGLNL
3. TaABCC13-5A EWLCLRMELLSTFVFAFCMAILVSFPPGTIEPSMAGLAVTYGLNL
4. OsABCC13 EWLCLRMELLSTFVFAFCMAILVSFPPGTIEPSMAGLAVTYGLNL
5. ZmABCC4 EWLCLRMELLSTFVFAFCMAILVSFPPGTIEPSMAGLAVTYGLNL
6. GmABCC1 EWLCLRMELLSTFVFAFCMVLLVSFPRGSIDPSMAGLAVTYGLNL
7. GmABCC2 EWLCLRMELLSTFVFAFCMVLLVSFPRGSIDPSMAGLAVTYGLNL
8. PvABCC1 EWLCLRMELLSTFVFAFCMVLLVSFPRGTIDPSMAGLAVTYGLNL
9. GmABCC3 EWLCLRMELLSTFVFAFCLVLLVSLPHGSIDPSMAGLAVTYGLNL
10. PvABCC2 EWLCLRMELLSTFVFAFCLVLLVSLPHGSIDPSMAGLAVTYGLNL

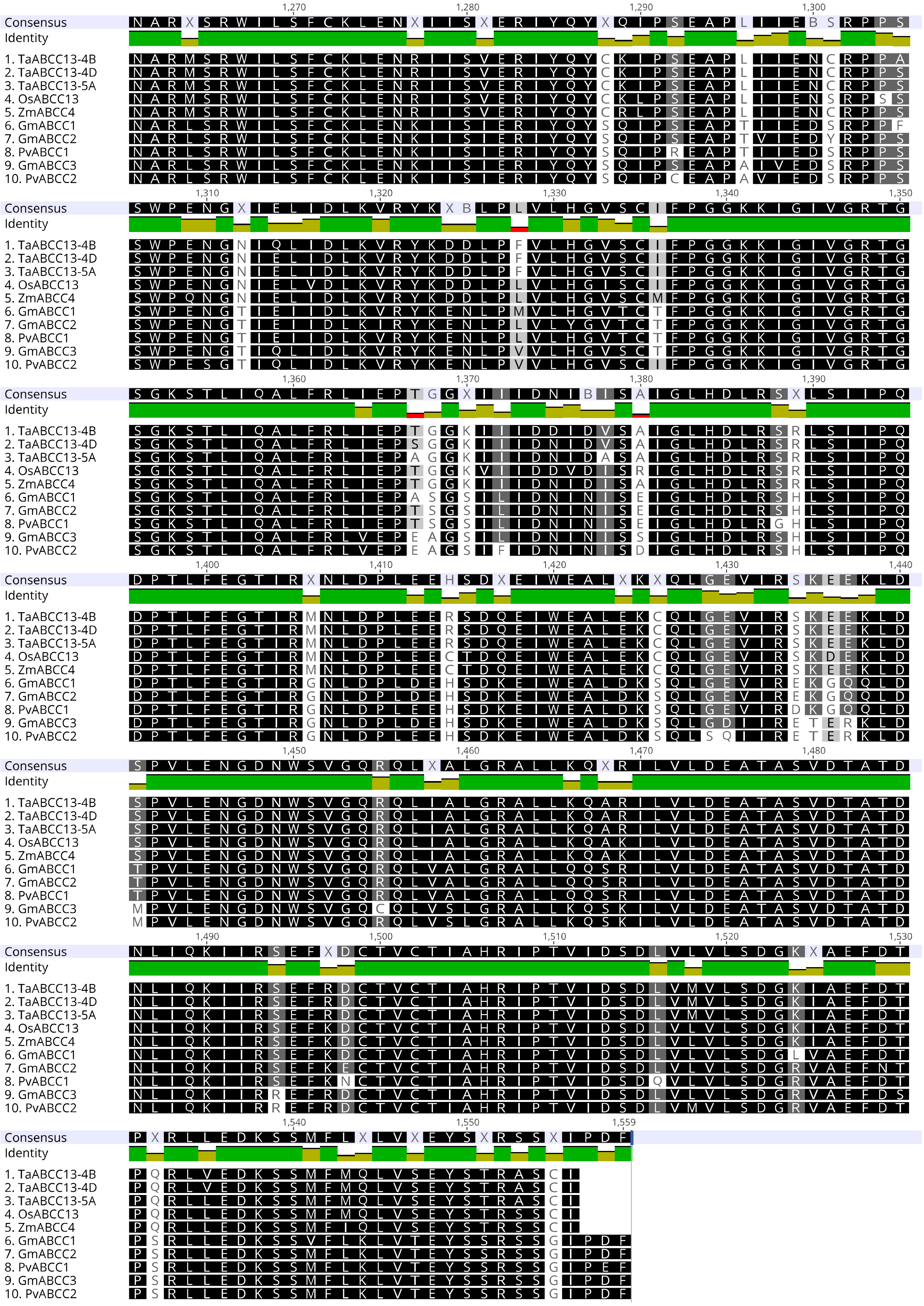

Figure S1. PA-MRP proteins alignment. See Table 1 for the correspondence with genes accession numbers.
The Clustal W alignment (cost matrix Blosum, gap open cost 10, gap extend cost 0.1) of Geneious 11.0.2 software was used.
