# Supplementary material for: Phytic Acid and Transporters: What Can We Learn from low phytic acid Mutants?
Source: Plants (Basel). 2020 Jan 5;9(1):69. doi: 10.3390/plants9010069 (PMC7020491; doi:10.3390/plants9010069)
Supplement: Supplementary file 1 [file plants-09-00069-s001.zip › Figure S3.pdf]

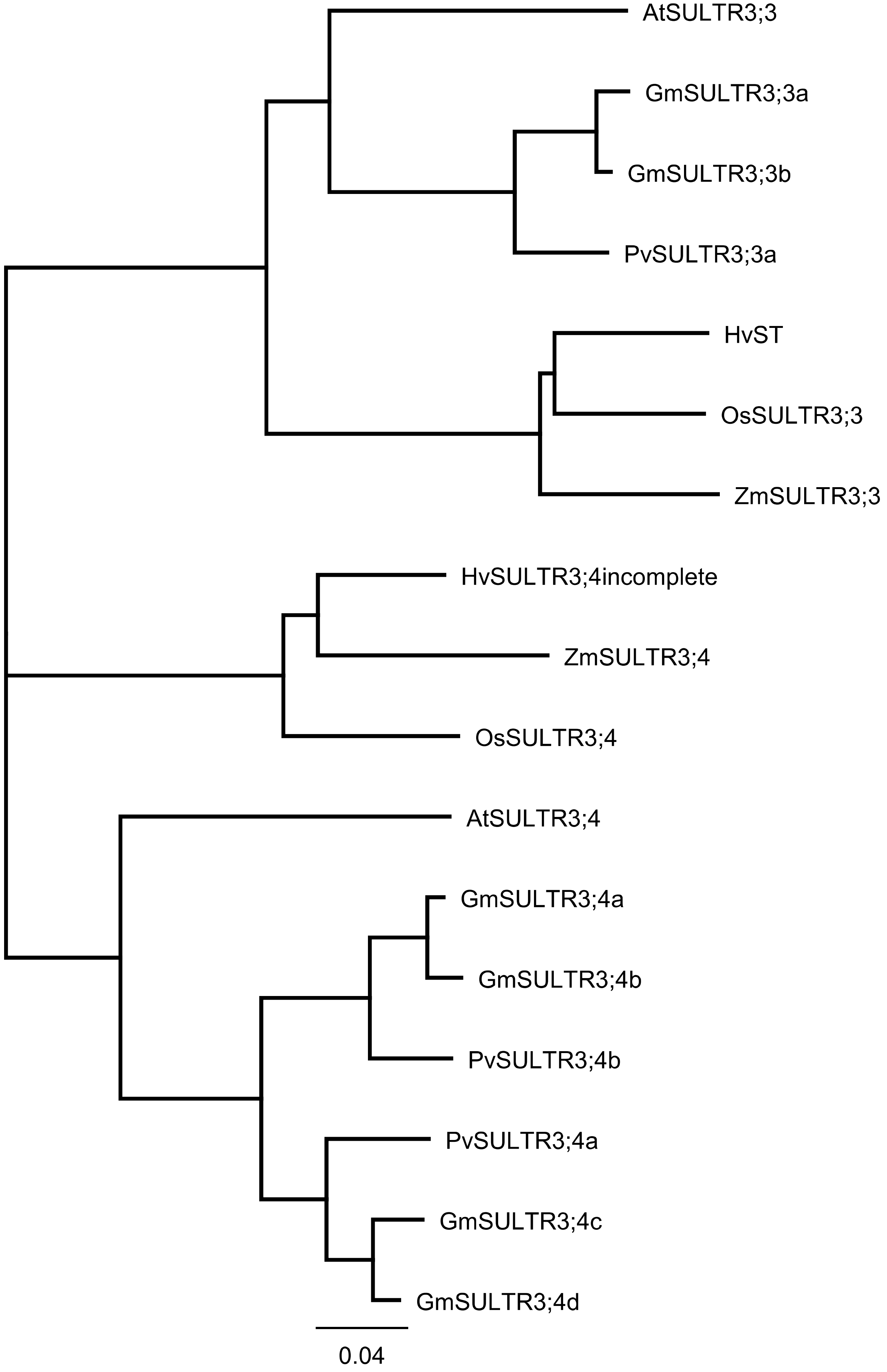

Figure S3. Phylogenetic tree of SULTR3;3 and SULTR3;4 proteins, listed in Table 2. Phylogenies were constructed as described in Figure S2.
