# Supplementary material for: Phytic Acid and Transporters: What Can We Learn from low phytic acid Mutants?
Source: Plants (Basel). 2020 Jan 5;9(1):69. doi: 10.3390/plants9010069 (PMC7020491; doi:10.3390/plants9010069)
Supplement: Supplementary file 1 [file plants-09-00069-s001.zip › Figure S5.pdf]

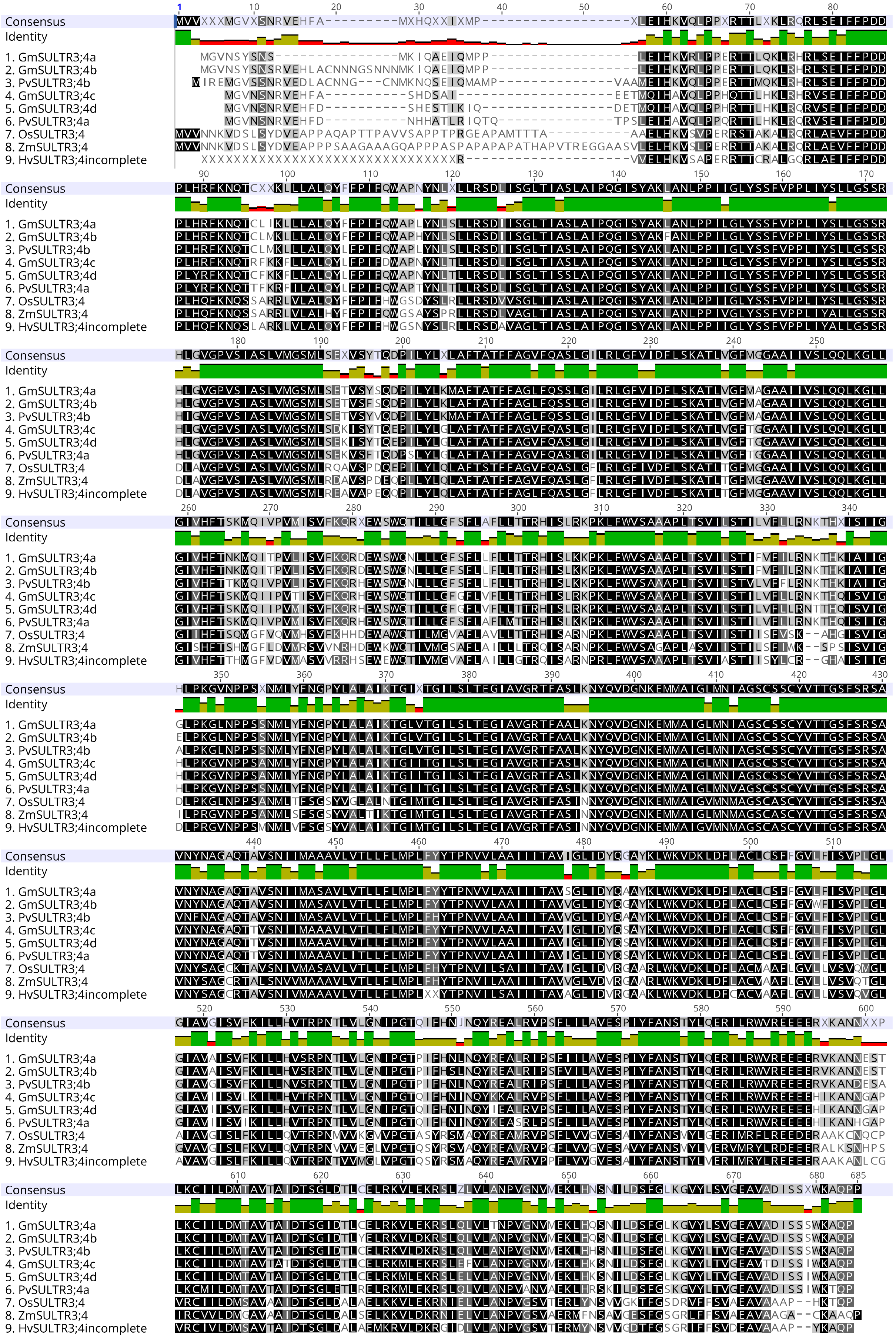

Figure S5. SULTR3;4 proteins alignment. See Tables 1 and 2 for the correspondence with genes accession numbers. The method used is described in Figure S1 legend.
