# Supplementary material for: Phytic Acid and Transporters: What Can We Learn from low phytic acid Mutants?
Source: Plants (Basel). 2020 Jan 5;9(1):69. doi: 10.3390/plants9010069 (PMC7020491; doi:10.3390/plants9010069)
Supplement: Supplementary file 1 [file Figure S4.pdf › Figure S4.pdf]

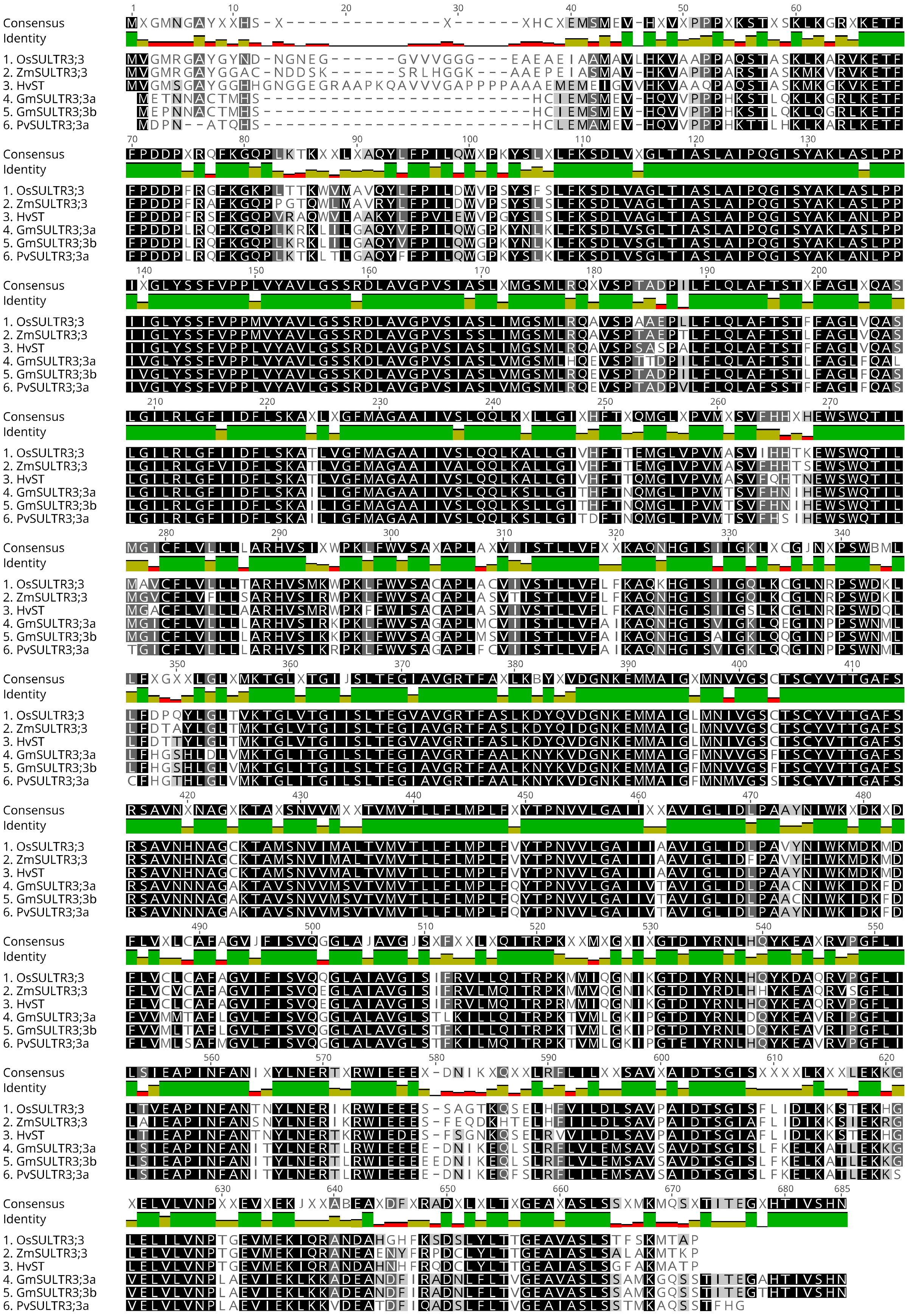

Figure S4. SULTR3;3 proteins alignment. See Tables 1 and 2 for the correspondence with genes accession numbers. The method used is described in Figure S1 legend.
